# Manipulating Cellular pH Suggests Novel Anticancer Therapy

**DOI:** 10.1371/journal.pbio.0050010

**Published:** 2006-12-19

**Authors:** Liza Gross

In the 1920s, when the genetic theory of cancer was taking root, German biochemist Otto Warburg (winner of the 1931 Nobel prize in physiology or medicine) proposed that abnormal energy metabolism caused cancer. Cells can generate energy either through oxidative respiration in mitochondria or through glycolysis in the cytoplasm. Warburg showed that tumors have an acidic extracellular environment, and he argued that a switch from oxidative respiration to glycolysis—which produces lactic acid—sets the cell down the path to cancer.

Though most cancer researchers think this glycolytic shift is a by-product rather than a cause of cancer, Warburg’s work stimulated interest in the possibility that there was some kind of link between pH and cancer. And now, a new study by Rui Zhao, Denis Alexander, and colleagues shows why manipulating the intracellular pH of tumor cells may indeed turn out to be a promising anticancer strategy.

Cells typically respond to genetic injury by triggering a carefully regulated program called apoptosis that kills cells with aberrant DNA so they don’t persist and spawn malignant progeny. In healthy cells, “pro-survival” proteins like Bcl-xL keep “pro-apoptotic” proteins like Bim in check. Recent work by other groups showed that DNA damage rapidly “deamidates” asparagine (Asn) amino acid residues in Bcl-xL —that is, removes their amide functional groups—converting Asn into aspartic acid (Asp). Now regarded as one of the most common post-translational protein modifications, Asn deamidation can substantially alter a protein’s function.

The finding that DNA damage can induce Asn deamidation suggests that it is a regulated event, challenging the established view that deamidation rates are predetermined only by a protein’s structural properties. These studies also suggested that damage-induced deamidation causes apoptosis, based on observations that deamidated Bcl-xL failed to inhibit pro-apoptotic Bim proteins in cells that died. But this link between Bcl-xL deamidation and apoptosis was called into question when additional experiments showed that deamidated Bcl-xL proteins could block pro-apoptotic proteins, and thus apoptosis, after all.

Against this background, Zhao et al. investigated the biochemical path from DNA damage to Bcl-xL deamidation along with its physiological consequences. Based on their own previous work—which showed that an oncogenic enzyme inhibited Bcl-xL deamidation, promoted Bcl-xL–Bim binding, and allowed DNA-damaged developing T cells (called thymocytes) to survive in a mouse model of T cell lymphoma—the researchers concluded that Bcl-xL deamidation is a “critical switch” in transforming T cells. And they suspected that the conflicting findings about deamidated Bcl-xL’s role in apoptosis reflected the fact that when Asn amino acid residues are deamidated, it’s mainly an isomer of Asp that results (iso-Aspartate or iso-Asp), not Asp itself.

Zhao et al. first determined that DNA-damage–triggered Bcl-xL deamidation is the cause, rather than the result, of apoptosis. Working in mouse thymocytes, they showed that inhibiting apoptosis in DNA-damaged cells did not inhibit Bcl-xL deamidation, which occurred 3 to 6 hours after DNA injury and proceeded along with increased apoptosis.

**Figure:**
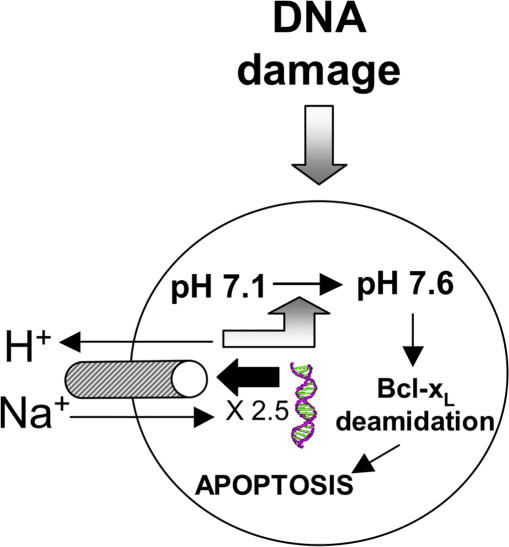
A novel signaling pathway triggered by DNA damage leads to the upregulation of the NHE-1 antiport, increased intracellular pH, Bcl-x_L_ deamidation, and finally apoptosis.

The model that Bcl-xL deamidation triggers apoptosis assumes that Bcl-xL promotes survival by sequestering the pro-apoptotic proteins. Since this activity represents the critical molecular link between DNA damage and apoptosis, the authors used a series of cellular and biochemical approaches to test this assumption. Working with oncogene-expressing but precancerous mouse thymocytes that resist deamidation, the authors found both Bim and another pro-apoptotic protein, Puma, bound to nonmodified Bcl-xL. Normal cells, however, could not sequester the pro-apoptotic proteins after DNA damage, indicating that deamidated Bcl-xL can’t bind them.

They tested this conclusion by exposing purified recombinant Bcl-xL to alkaline conditions that induced partial Bcl-xL deamidation and produced three Bcl-xL species, each with different deamidation profiles. Only one of these forms—in which Asn converts to iso-Asp—prevents the sequestration of the pro-apoptotic proteins. This isomerized form disrupts Bcl-xL’s structure enough to prevent it from binding Bim or Puma. It’s well known that iso-Asp has important structural, and thus functional, implications for a broad range of proteins, because it puts a ‘kink’ into the amino acid backbone of proteins that Asp doesn’t.

Having observed that elevated pH speeds up Asn deamidation rates, the authors investigated the possibility that alkalinization induces deamidation in the thymocytes. While pH levels did not change in the precancerous cells, it rose in response to DNA damage in nonmutant cells—an increase associated with significant Bcl-xL deamidation. In fact, artificially increasing the alkalinity of the DNA-damaged precancerous thymocytes—normally resistant to deamidation—triggered deamidation. Even in thymocytes without DNA damage, alkalinization led to considerable Bcl-xL deamidation along with increased apoptosis. Importantly, precancerous thymocytes artificially engineered to express Bcl-xL species that can sequester Bim survived, despite enforced alkalinization.

But what regulates the rise in pH inside the cell? DNA damage triggers increased production of a plasma membrane protein known as the Na/H exchanger (NHE-1), or antiport (because it exchanges extracellular sodium ions with cytoplasmic hydrogen ions), which allows for a higher efflux of hydrogen ions, thereby raising the intracellular pH.

Altogether, these results chart the molecular path to Bcl-xL deamidation: DNA damage triggers a 2- to 3-fold increase in the production of NHE-1, allowing an efflux of hydrogen ions, which raises intracellular pH. Alkalinization, in turn, deamidates two Bcl-xL Asn residues into iso-Asp, which alters the pro-survival protein’s shape, preventing it from binding to and sequestering pro-apoptotic proteins, leading to apoptosis.

To determine whether the novel signaling pathway they had elucidated in mouse cells might have relevance to human cancer cells, the authors also studied cells from patients with chronic lymphocytic leukemia (CLL). They found that when the intracellular pH was raised artificially, both Bcl-xL deamidation and apoptosis resulted.

Though Warburg’s metabolic theory of cancer never gained traction, his work highlighted a possible connection between pH and cancer—and now, 70 years later, that link is receiving experimental support. Many different tumor types appear to use Bcl-xL to bypass apoptosis, rendering some cancer cells resistant to therapy. This suggests that inducing alkalinization—possibly by enhancing NHE-1 expression or increasing its activity—to promote Bcl-xL deamidation and thus apoptosis may prove an effective strategy to treat a range of cancers.

